# Genome-Wide Correlation of DNA Methylation and Gene Expression in Postmortem Brain Tissues of Opioid Use Disorder Patients

**DOI:** 10.1093/ijnp/pyab043

**Published:** 2021-07-02

**Authors:** Andi Liu, Yulin Dai, Emily F Mendez, Ruifeng Hu, Gabriel R Fries, Katherine E Najera, Shan Jiang, Thomas D Meyer, Laura Stertz, Peilin Jia, Consuelo Walss-Bass, Zhongming Zhao

**Affiliations:** 1 Department of Epidemiology, Human Genetics and Environmental Sciences, School of Public Health, The University of Texas Health Science Center at Houston, Houston, TX, USA; 2 Center for Precision Health, School of Biomedical Informatics, The University of Texas Health Science Center at Houston, Houston, TX,USA; 3 Louis A. Faillace, MD, Department of Psychiatry and Behavioral Sciences, McGovern Medical School, The University of Texas Health Science Center at Houston, Houston, TX,USA; 4 MD Anderson Cancer Center UTHealth Graduate School of Biomedical Sciences, Houston, TX, USA

**Keywords:** Astrocyte, DNA methylation, integrative genomic analysis, opioid use disorder, postmortem brain

## Abstract

**Background:**

Opioid use disorder (OUD) affects millions of people, causing nearly 50 000 deaths annually in the United States. While opioid exposure and OUD are known to cause widespread transcriptomic and epigenetic changes, few studies in human samples have been conducted. Understanding how OUD affects the brain at the molecular level could help decipher disease pathogenesis and shed light on OUD treatment.

**Methods:**

We generated genome-wide transcriptomic and DNA methylation profiles of 22 OUD subjects and 19 non-psychiatric controls. We applied weighted gene co-expression network analysis to identify genetic markers consistently associated with OUD at both transcriptomic and methylomic levels. We then performed functional enrichment for biological interpretation. We employed cross-omics analysis to uncover OUD-specific regulatory networks.

**Results:**

We found 6 OUD-associated co-expression gene modules and 6 co-methylation modules (false discovery rate <0.1). Genes in these modules are involved in astrocyte and glial cell differentiation, gliogenesis, response to organic substance, and response to cytokine (false discovery rate <0.05). Cross-omics analysis revealed immune-related transcription regulators, suggesting the role of transcription factor-targeted regulatory networks in OUD pathogenesis.

**Conclusions:**

Our integrative analysis of multi-omics data in OUD postmortem brain samples suggested complex gene regulatory mechanisms involved in OUD-associated expression patterns. Candidate genes and their upstream regulators revealed in astrocyte, and glial cells could provide new insights into OUD treatment development.

**Significance Statement**

Mortality associated with opioid use disorder (OUD) is increasing in the United States. However, the molecular pathogenesis of OUD is still mostly elusive, hindering treatment development. Thus, it is crucial to investigate the complex pathophysiology of OUD using multi-omics approaches. In this study, we conducted an integrative transcriptome and DNA methylation analysis using data generated from OUD postmortem brain samples and controls. Our results revealed key biological processes dysregulated in OUD, including astrocytic processes, neurogenesis, cytokine response, and glial cell differentiation as well as transcription factor regulation. Our study revealed a complex relationship between DNA methylation, transcription factor regulation, and gene expression, reflecting the epigenetic heterogeneity of OUD. These findings may help develop treatment and prevention strategies for OUD.

## Introduction

During the past few decades, opioids have been increasingly prescribed for non-cancer pain in the United States. However, opioid medication use puts patients at risk for opioid use disorder (OUD), which affects over 2 million individuals and causes nearly 50 000 deaths per year in the United States (National Institute on Drug Abuse, 2021). This causes heavy societal and economic burdens (Chang et al., 2018). According to the DSM-V criteria, OUD is defined as a problematic pattern of opioid use leading to clinically significant impairment or distress (American Psychiatric Association, 2013). OUD may be caused by a combination of environmental, behavioral, and biological factors, although the precise molecular mechanisms of OUD remain elusive.

Previous studies have shown significant genetic and epigenetic alterations between OUD patients and controls. Twin and family studies indicate genetic inheritance contributes to OUD, and heritability estimates range from 40% to 60% (Merikangas et al., 1998; Tsuang et al., 1998; Kendler et al., 2003; Sun et al., 2012). Genetic variations in the opioid receptor mu 1 gene (*OPRM1*) and other genes (*KCNC1*, *COMT*, *ESR1*) are also associated with OUD (Gelernter et al., 2014; De Gregori et al., 2016; Smith et al., 2017). Several case-control studies have revealed differentially expressed genes in the OUD brains as well as enrichment of dopaminergic and opioid receptor signaling pathways (Przewlocki, 2004; Saad et al., 2019). Moreover, OUD and opioid exposure are associated with epigenetic modifications, including alterations in DNA methylation, histone modifications, and microRNA expression (Migliore and Coppede, 2009; Browne et al., 2020). In particular, DNA methylation is a complex gene regulatory mechanism whereby cytosine hypermethylation canonically induces a condensed chromatin state and downregulates transcription of the target gene at the promoter region (Bird and Wolffe, 1999; Wang et al., 2015). *OPRM1* is hypermethylated in the blood of short-term and long-term opioid users (Chorbov et al., 2011; Sandoval-Sierra et al., 2020). DNA methylation can be studied using microarray or bisulfate methylation sequencing. Many complex roles of DNA methylation have been documented, such as its regulation in mammalian development and differentiation (Suzuki and Bird, 2008). However, further studies are needed to investigate how epigenetic alterations contribute to the development or consequences of OUD.

While traditional bioinformatics studies focus on correlating single-modality transcriptomic or epigenetic signatures with disease phenotype, multi-omics approaches allow a comprehensive assessment of gene regulatory relationships. Such cross-domain analysis strategies can reduce false discoveries as well (Jia et al., 2019). Until recently, few multi-omics studies have been conducted in OUD to explore complex regulatory mechanisms at the genomic and epigenomic levels (Crist et al., 2019), and even fewer studies use data generated from human tissue. In this study, we systematically characterized DNA methylation and transcriptomic gene networks in postmortem brain samples of 22 OUD subjects and 19 non-psychiatric controls. Specifically, we used weighted gene co-expression network analysis (WGCNA) to identify OUD-associated differentially co-expressed or co-methylated network modules (Langfelder and Horvath, 2008). Subsequent gene set enrichment analyses identified dysregulated biological processes within these identified network modules. Furthermore, we conducted cross-omics analyses of transcriptome and DNA methylome data to unveil convergent pathways and gene regulatory patterns underlying OUD pathogenesis.

## Materials and Methods

### Samples

Postmortem brain tissues were obtained from The University of Texas Health Science Center at Houston (UTHealth) Brain Collection in collaboration with the Harris County Institute of Forensic Science with institutional review board approval. Demographic information, autopsy and toxicology reports, and medical and psychiatric notes were collected for each patient. A detailed psychological autopsy was obtained for each subject by interviewing the next of kin, where information regarding psychiatric clinical phenotypes (such as evidence of depression, mania, psychosis), age of onset of drug use, types of drugs used, smoking and drinking history, and co-morbidities was collected. OUD or control consensus diagnosis was reached for each subject according to DSM-V criteria by an independent panel of 3 trained clinicians after review of all available case information.

Brains were collected from 22 OUD and 19 non-psychiatric control subjects (Table 1). Of them, samples of 22 OUD subjects and 18 controls were used to generate RNA sequencing data, and samples of 19 OUD subjects and 11 controls were used to generate DNA methylation data. Postmortem interval (PMI) was calculated from the time of death until tissue preservation. On brain receipt, the right hemisphere was coronally sectioned, immediately frozen, and stored at −80º C. Brodmann area 9 (BA9) was defined within the prefrontal cortex between the superior frontal gyrus and the cingulate sulcus. Dissections of BA9 were obtained using a 4-mm cortical punch, yielding approximately 100 mg of tissue. Then 100–200 mg of the cerebellum was dissected to measure cerebellar pH, as previously described (Monoranu et al., 2009).

**Table 1.** Characteristics of OUD Patients and Non-psychiatric Controls

	Gene expression data (total n = 40)			Methylation data (total n = 30)		
	OUD patients	Controls	*P* value	OUD patients	Controls	*P* value
Samples, n	22 (55.00%)	18 (45.00%)		19 (63.33%)	11 (36.67%)	
Age, mean (SE)	38.00 (2.72)	53.06 (3.68)	.002^*a*^	38.21 (2.99)	47.18 (5.01)	.143^*a*^
Gender, n (%)						
Male	11 (50.00%)	16 (88.90%)	.016^*b*^	10 (52.63%)	10 (90.91%)	.049^*b*^
Female	11 (50.00%)	2 (11.10%)		9 (47.37%)	1 (9.09%)	
Race/ethnicity, n						
White	18 (81.80%)	10 (55.60%)	.129^*b*^	15 (78.95%)	6 (54.55%)	.211^*b*^
Hispanic	0 (0.00%)	2 (11.10%)		0 (0.00%)	1 (9.09%)	
African American	4 (18.10%)	5 (27.80%)		4 (21.05%)	3 (27.27%)	
Asian	0 (0.00%)	1 (5.60%)		0 (0.00%)	1 (9.09%)	
PMI, mean (SE)	26.57(2.12)	29.66 (1.80)	.273^*a*^	27.89 (2.28)	27.90 (2.52)	.997^*a*^
RIN, mean (SE)	7.03 (0.21)	7.01 (0.17)	.938^*a*^	6.98 (0.24)	7.05 (0.24)	.835^*a*^
pH, mean (SE)	6.57 (0.04)	6.57 (0.06)	.987^*a*^	6.59 (0.05)	6.52 (0.09)	.519^*a*^

Abbreviations: OUD, opioid use disorder; PMI, postmortem interval; RIN, RNA integrity number.

^*a*^Comparison between OUD patients and controls by *t* test.

^*b*^Comparison between OUD patients and controls by Fisher’s exact test.

### RNA Preparation and Sequencing

**Figure 1.**
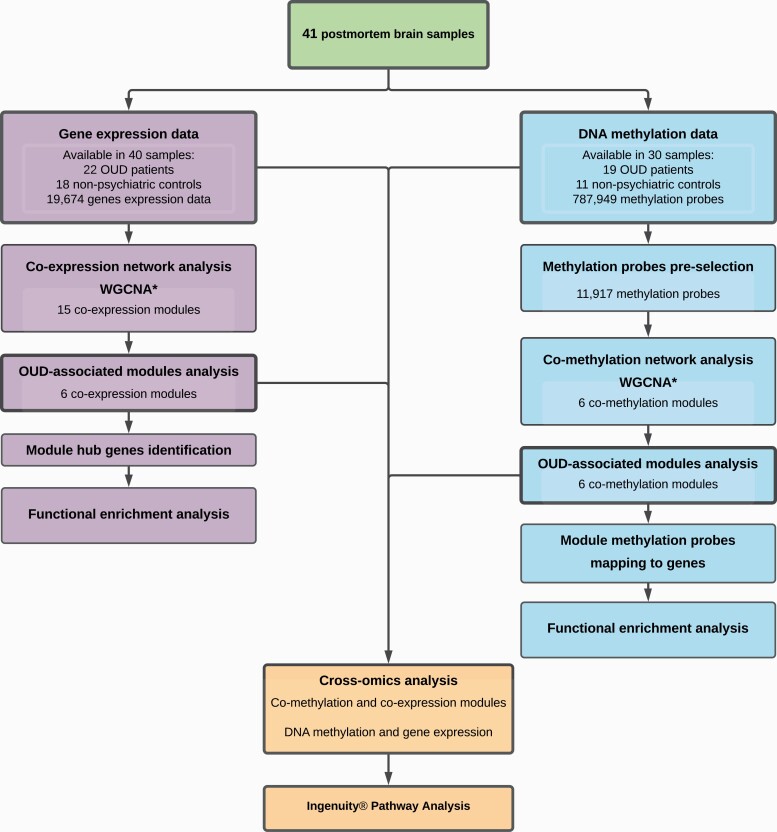
Workflow of the study. WGCNA, weighted gene co-expression network analysis; OUD, opioid use disorder.

The general project pipeline is shown in Figure 1. A total 22 OUD subjects and 18 controls were selected for RNA sequencing. RNA of each subject was extracted from 50 mg of BA9 tissue using RNeasy Plus Mini kits (QIAGEN Inc., Hilden, Germany). RNA integrity number (RIN) was measured to assess RNA quality (Agilent Bioanalyzer 2100 system, Agilent Technologies, Santa Clara, CA, USA). Then 1 μg RNA per sample was used for mRNA library construction using NEBNext Ultra RNA Library Prep Kit (Illumina Inc., San Diego, CA). Paired-end sequencing reads (150 bp) were generated on an Illumina HiSeq2000 platform (Q30 > 80%) at Novogene Bioinformatics Institute (Chula Vista, CA, USA).

Sample sequencing quality was evaluated using FastQC (Simon, 2010). We used STAR (Dobin et al., 2013) for read mapping and annotation based on Genome Reference Consortium Human Build 37 (GRCh37) and obtained read count per gene for differential gene expression analysis. After mapping the fastq files to the reference genome, unique mapping rates were found to be between 82.5% and 90.7% (average: 88.2%), which qualified data for downstream analysis.

### Differentially Expressed Gene Analysis

The R package *DEseq2* was used to identify differentially expressed genes (DEGs) (Love et al., 2014). The input matrix contained un-normalized read counts for each gene in each sample. Genes were excluded if they had read counts of 0 in more than 50% of samples or if the variance across all samples was less than 1. After these filtrations, 20 030 genes were selected for DEG analysis. In addition to control/OUD diagnosis, the differential expression model included the following human tissue covariates: age, sex, PMI, RIN, and cerebellar pH. Due to the small sample size, continuous covariates were factored into bins based on guidelines from the author (Love et al., 2014), denoted by Age_N, PMI_N, RIN_N, and pH_N. Specifically, age was categorized by every 10 years; PMI, RIN, and pH were each divided into 5 groups with equal interval sizes.

The following formula was used for differential gene expression:


$$\begin{array}{l} design\_formula_{DEG}∼Diagnosis+Sex+Age_{N}+PMI_{N}+RIN_{N}+pH_{N} \end{array}$$

Benjamini-Hochberg (BH) procedure (Benjamini and Hochberg, 1995) was applied for multiple test correction, with false discovery rate (FDR) set to 0.05. Log2 fold changes of gene expression between OUD and control subjects were generated from the DEG analysis and were used in the subsequently cross-omics analysis.

### Methylation Data Generation and Processing

DNA from 19 OUD subjects and 11 non-psychiatric controls was isolated from brain tissues using the DNeasy Blood and Tissue Kit (QIAGEN Inc.). After quantification on a NanoDrop (Thermo Fisher Scientific, Waltham, MA, USA), 500 ng of DNA was bisulfite-converted with the EZ DNA Methylation Kit (Zymo Research) and was processed for hybridization to the Infinium MethylationEPIC kit (Illumina Inc., San Diego, CA, USA). Signal was detected in an iScan microarray reader (Illumina Inc.) according to the manufacturer’s instructions, generating raw IDAT files for downstream analyses.

To assess the sample quality, the average detection *P* value was obtained using the R package *minfi* (version 1.30.0) (Aryee et al., 2014). Specifically, the background signal was estimated using negative control probes, and the detection *P* value was calculated by comparing the total signal for each probe with the background signal. The average detection *P* value across probes was then calculated for each sample. All samples were of high quality (*P* < .05) and were kept for the following analysis.

Probes were removed that met the following parameters: (1) probes with detection *P* > .01 in 50% of the samples; (2) probes mapped on the X or Y chromosomes; (3) probes known to have common single-nucleotide polymorphisms at the CpG sites; and (4) probes that mapped to multiple locations in the human genome and therefore may be cross-reactive. The original array contained 1 051 539 probes. A total 263 590 probes were filtered out, leaving 787 949 probes for the downstream analysis.

We normalized the intensity Y_i__methy and Y_i__unmethy of each probe *i* into M_i_ using the following formula:

$M_{i}=log_{2}[max(Y_{i}\_methy,0)/max(Y_{i}\_unmethy,0)]\;$ (Du et al., 2010).

### Differentially Methylated Probes (DMP) Analysis

The DMP analysis was conducted on the normalized probe intensity M using the R package *limma* (version 3.44.3) (Ritchie et al., 2015). In addition to OUD/control diagnosis, the regression model adjusted for the following human tissue covariates: sex, age, PMI, and pH. The designed formula is shown below:


$$\begin{array}{l} design\_\;formula_{DMP}∼Diagnosis+Sex+Age+PMI+pH \end{array}$$

The *P* value and log2 fold change of methylation probes between 2 groups were generated from DMP analysis and used in the cross-omics analysis.

To balance the probe numbers and variance in co-methylation network analysis, DMPs were filtered based on nominal significance and probe location. DMPs with nominal *P* < .05 and location at transcriptional start sites (TSSs) were used in weighted co-methylation network analysis. Out of 787 949 normalized methylation probes, 11 917 methylation probes met these criteria for further analysis.

### Co-expression/Co-methylation Network Analysis

For the co-expression and co-methylation network analyses, gene expression data were normalized using variance-stabilizing transformation (Durbin et al., 2002), and methylation data (beta-value) were normalized to the M-value described above. In co-expression network analysis, 19 389 normalized genes and 11 917 normalized methylation probes were used after filtering out genes with missing values. WGCNA R package (WGCNA version 1.70–3, R version 4.0.2) was used to analyze the normalized data. Detailed methodology can be found in the original publication (Langfelder and Horvath, 2008). The gene co-expression network was constructed using a soft threshold power, allowing the network to approximate scale-free topology (scale-free fit signed R^2^ > 0.8). For co-methylation network construction, an empirical parameter of 14 was used for the power adjacency function based on guidelines from the authors (Langfelder and Horvath, 2014).

Network modules of co-expressed genes and co-methylation probes were detected using the *WGCNA* package *blockwiseModules* function with the settings:

*networkType = “signed,” minModuleSize = 30, corType = “bicor,”* and *deepSplit = 3*.

Each network module was assigned a unique color by the package. OUD-associated co-expression and co-methylation modules were identified using linear regression between module eigengenes, defined as the first principal component of each module, and OUD diagnosis trait after controlling for the following covariates: age, gender, ethnicity, sample PMI, RIN, and pH. Significantly correlated OUD modules were determined by BH-corrected *P*-value with FDR (*P*_FDR_) < .1, which corrected for the total number of modules in each analysis (Benjamini and Hochberg, 1995).

### Identification of Network Hub Genes of Co-expression Modules

For each co-expression module, hub genes were defined as genes highly connected with the other genes in the module, representing a potentially critical role in the module network (Langfelder and Horvath, 2008; Sun et al., 2011). Specifically, hub genes were selected as genes with module membership ≥0.8 and gene trait significance ≥0.2 within each co-expression module (Langfelder and Horvath, 2016).

### Functional Enrichment Analysis of Module Genes

To explore functional enrichment of hub genes in co-expression modules and annotated genes in co-methylation modules, the Database for Annotation, Visualization and Integrated Discovery (DAVID) (Huang et al., 2007) was used. Functional annotation tool (FAT) was used to survey significant Gene Ontology Biological Processes (BP), using all human protein-coding genes as the background. BP-FAT with BH corrected *P* value with FDR (*P*_FDR_) < .05 was considered significant (Benjamini and Hochberg, 1995).

### Cross-omics Analysis of Transcriptomic and Methylomic Data

The network module level and the gene level cross-omics analyses were conducted to integrate DNA methylation and gene expression analysis. At the network module level, a hypergeometric test was used to measure overrepresented genes in OUD-associated co-methylation modules and hub genes in co-expression modules of interest (Figure 1). Fisher’s exact test with raw *P* < .05 was used to examine if co-methylation modules and co-expression modules were overlapped significantly. Functional enrichment analysis of BP-FAT terms using DAVID as described above (*P*_FDR_ < .05) was then performed on overlapping genes for biological interpretation.

At the gene level, we examined global correlations between DNA methylation changes and gene expression by including all available DNA methylation and gene expression data (Figure 1). A total 787 949 methylation probes were mapped to 16 986 genes according to the Illumina annotation file. Then, mapped genes were plotted and analyzed using gene expression log2 fold changes from DEG analysis and DNA methylation log2 fold changes from DMP analysis. For genes that could be mapped by multiple methylation probes, the median log2 fold change values of all mapped methylation probes for that gene were assumed to represent the overall level of methylation changes. Four gene lists were obtained representing high levels of variation in DNA methylation (|DNA methylation log2 fold changes| ≥ 0.2) and gene expression (|gene expression log2 fold changes| ≥ 1).

### Ingenuity Pathway Analysis (IPA)

Canonical pathways and gene regulatory relationships were further explored using IPA (version 62 089 861, QIAGEN Inc.). Log2 fold change of genes in overlapping modules from the co-expression/co-methylation cross-omics analysis were input for IPA canonical pathway analysis. Significantly activated canonical pathways were defined as raw *P* < .05 and z-score > 0 (Kramer et al., 2014).

IPA analyses were also used to infer the upstream transcription regulators from genes with highly variable DNA methylation (|DNA methylation log2 fold changes| ≥ 0.2) and gene expression (|gene expression log2 fold changes| ≥ 1). Specifically, we used the expression log2 fold change of those genes as the input of IPA. We defined the overrepresented transcription factors with |activation z-score| > 1.96 and raw *P* < .01 based on the tutorial from IPA (Kramer et al., 2014).

## Results

### Subjects and Brain Samples

Table 1 summarizes the characteristics of human subjects. We generated gene expression data in 97.56% (40/41) of the subjects and methylation data in 73.17% (30/41) of subjects. A total of 19 OUD subjects and 10 non-psychiatric controls had both gene expression and methylation data. No significant differences were observed between the OUD subjects and controls in terms of PMI, RIN, or cerebellar pH for the gene expression or methylation cohort (*P* > .05). The OUD subjects were significantly younger than the controls (*P* = .002) in the gene expression cohort but not in the methylation cohort (*P* = .142). The OUD group contained more female subjects than the control group, and subjects were predominately white in both datasets. All covariates (PMI, cerebellar pH, age, gender, and RIN for RNA-seq data) were adjusted for subsequent analyses to eliminate potential bias.

### Co-expression Network Analysis

**Figure 2.**
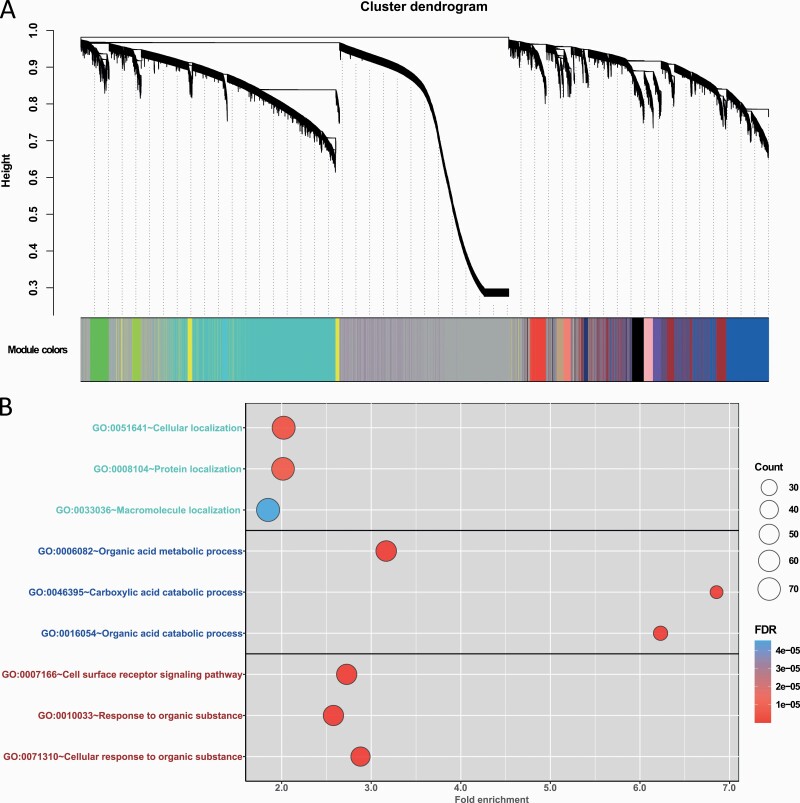
Weighted co-expression network and functional enrichment analysis of hub genes of co-expression modules. (A) Dendrogram of all expressed genes clustered based on a dissimilarity measure by Weighted gene co-expression network analysis (WGCNA). Co-expression modules turquoise, blue, and brown, as annotated by WGCNA, contained the most genes. (B) Top 3 enriched Gene Ontology (GO) Biological Process terms for co-expression module turquoise, blue, and brown. FDR, false discovery rate.

Transcriptomic data were analyzed in 40 postmortem brain samples (18 OUD subjects and 22 controls) (Table 1). After normalization, 19 389 genes were used for co-expression analysis. In total, 15 co-expression modules were detected with a median member size of 387 (Figure 2A). Six co-expression modules were significantly associated with the OUD diagnosis trait (*P*_FDR_ < .1) (Table 2). Among these modules, genes in the co-expression module turquoise (module size: 4376) and module cyan (#196) were downregulated in OUD subjects (negatively correlated with presenting of OUD trait), while genes in co-expression modules blue (#2629), brown (#1409), pink (#387), and purple (#308) were upregulated (Table 2). Of note, the color and module ID annotations were generated by the WGCNA tool.

**Table 2.** Linear Regression of Co-expression Module Eigengenes and OUD Trait

	Module size	Beta	SE	*P* value	*P*_FDR_
ME_turquoise_	4376	−0.18	0.06	3.43 × 10^–3^	.05
ME_blue_	2629	0.16	0.06	.01	.05
ME_brown_	1409	0.13	0.06	.03	.08
ME_yellow_	603	−0.08	0.07	.26	.41
ME_green_	526	−0.04	0.06	.54	.67
ME_red_	445	0.07	0.07	.29	.42
ME_black_	441	0.09	0.06	.11	.23
ME_pink_	387	0.16	0.06	.01	.05
ME_magenta_	356	0.04	0.07	.60	.69
ME_purple_	308	0.16	0.06	.01	.05
ME_greenyellow_	249	−0.01	0.07	.89	.89
ME_tan_	208	0.10	0.06	.14	.24
ME_salmon_	205	0.13	0.07	.06	.14
ME_cyan_	196	−0.14	0.06	.03	.08
ME_midnightblue_	124	−0.05	0.07	.47	.62

Abbreviations: FDR, false discovery rate; ME, module eigengene; OUD, opioid use disorder; SE, standard error.

All linear regressions analyses were conducted by controlling covariates, including age, gender, ethnicity, sample PMI, RIN, and pH.

### Functional Enrichment Analysis of Co-expression Module Hub Genes

Next, hub genes were identified in OUD-associated co-expression modules to investigate the critical functional roles of these key genes in each module. We identified 268 hub genes from co-expression module turquoise, 338 from module blue, 125 from module brown, 75 from module cyan, 8 from module pink, and 8 genes from module purple.

Hub genes in the 3 largest OUD associated co-expression modules (turquoise, blue, and brown) were used to find significant biological process terms by DAVID pathway enrichment tool (Figure 2B). The top 3 highly enriched biological processes of co-expression module turquoise hub genes (supplementary Table 1) were cellular localization (fold-enrichment = 2.03, *P*_FDR_ = 2.83 × 10^–6^), protein localization (fold-enrichment = 2.02, *P*_FDR_ = 4.35 × 10^–6^), and macromolecule localization (fold-enrichment = 1.85, *P*_FDR_ = 3.76 × 10^–5^). Co-expression module blue hub genes (supplementary Table 2) were highly enriched in organic acid metabolic process (fold-enrichment = 3.17, *P*_FDR_ = 5.31 × 10^−10^), carboxylic acid metabolic process (fold-enrichment = 6.86, *P*_FDR_ = 5.85 × 10^−10^), and organic acid catabolic process (fold-enrichment = 6.23, *P*_FDR_ = 1.05 × 10^–9^). Co-expression module brown hub genes (supplementary Table 3) were enriched in cell surface receptor signaling pathway (fold-enrichment = 2.73, *P*_FDR_ = 5.78 × 10^–9^), response to organic substance (fold-enrichment = 2.58, *P*_FDR_ = 2.49 × 10^–8^), and cellular response to organic substance (fold-enrichment = 2.88, *P*_FDR_ = 2.59 × 10^–8^).

Furthermore, we collected brain cell function–specific genes from a previous study (Goudriaan et al., 2014) and conducted a hypergeometric test to identify brain cell type signatures that were associated with the hub genes in co-expression modules. The hub genes in co-expression module blue were found to be strongly associated with astrocyte transport, trafficking, and metabolism related functions (supplementary Figure 1). These findings suggested that the blue module might be enriched in astrocytic functions.

### Co-methylation Network Analysis

DNA methylomic data were analyzed in postmortem brain samples of 19 OUD subjects and 11 controls (Table 1). To balance probe numbers and variance in co-methylation network analysis, methylation probes were filtered based on DMP analysis significance (at nominal *P* < .05) and probe location at TSSs. A total 11 917 methylation probes were used in the weighted co-methylation analysis.

**Figure 3.**
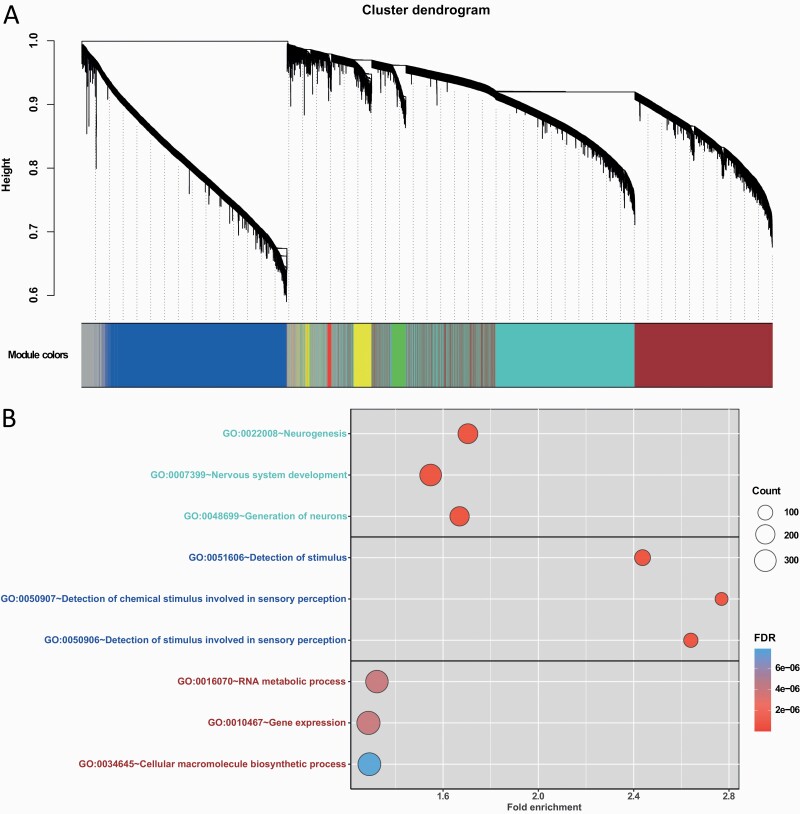
Weighted co-methylation network and functional enrichment analysis of uniquely mapped genes of co-methylation modules. (A) Dendrogram of pre-selected methylation probes clustered based on a dissimilarity measure by Weighted gene co-expression network analysis (WGCNA). Co-methylation modules turquoise, blue, and brown contained the most genes. (B) Top 3 enriched Gene Ontology (GO) Biological Process terms for co-methylation module turquoise, blue, and brown. FDR, false discovery rate.

Following the same procedure as the co-expression WGCNA, we performed weighted network analysis on methylation data. The methylation probes clustered into 6 modules with a median member size of 1782.5 probes (Figure 3A; Table 3). All 6 modules were significantly associated with the OUD diagnosis trait (*P*_FDR_ ≤ .1) (Table 3). Among the 6 co-methylation modules, module eigengenes of co-methylation module blue were positively associated with the OUD trait, indicating hypermethylated loci were congregated in this module. Thus, mapped gene transcription is likely repressed within co-methylation module blue. Inversely, module eigengenes of other co-methylation modules were negatively associated with the OUD trait, suggesting hypomethylated loci clustered in these modules, which would likely lead to upregulation of the mapped genes (Table 3).

**Table 3.** Linear Regression of Co-methylation Module Eigengenes and OUD Trait

	Module size	Uniquely mapped gene	Beta	SE	*P* value	*P*_FDR_
ME_turquoise_	3898	2012	−0.17	0.08	.04	.09
ME_blue_	3146	1860	0.15	0.08	.08	.09
ME_brown_	3135	1286	−0.14	0.08	.09	.09
ME_yellow_	430	204	−0.17	0.08	.06	.09
ME_green_	303	141	−0.18	0.09	.06	.09
ME_red_	76	36	−0.20	0.08	.02	.05

Abbreviations: FDR, false discovery rate. ME, module eigengene. OUD, opioid use disorder. SE, standard error.

All linear regressions analyses were conducted by controlling covariates, including age, gender, ethnicity, sample PMI, RIN, and pH.

### Functional Enrichment Analysis of Co-methylated Modules

Methylation probes in each module were mapped to genes according to the Illumina annotation file for subsequent functional enrichment analyses (Table 3). We observed enriched biological processes among the 3 largest co-methylation modules (turquoise, blue, brown). Figure 3B shows the top 3 results of significantly enriched pathways for uniquely mapped genes in co-methylation modules of interest. In module turquoise, 3898 methylation probes were mapped to 2012 unique genes. The top 3 highly enriched biological processes in co-methylation module turquoise (supplementary Table 4) included neurogenesis (fold-enrichment = 1.70, *P*_FDR_ = 2.39 × 10^–12^), nervous system development (fold-enrichment = 1.55, *P*_FDR_ = 2.39 × 10^–12^), and generation of neurons (fold-enrichment = 1.67, *P*_FDR_ = 2.20 × 10^−10^). The enriched biological processes for the 1860 uniquely mapped genes of co-methylation module blue (supplementary Table 5) included detection of stimulus (fold-enrichment = 2.43, *P*_FDR_ = 2.56 × 10^–15^), detection of chemical stimulus involved in sensory perception (fold-enrichment = 2.77, *P*_FDR_ = 2.99 × 10^–15^), and detection of stimulus involved in sensory perception (fold-enrichment = 2.64, *P*_FDR_ = 5.20 × 10^–15^). The 1286 uniquely mapped genes of co-methylation module brown (supplementary Table 6) were enriched in biological processes of RNA metabolic process (fold-enrichment = 1.32, *P*_FDR_ = 4.03 × 10^–6^), gene expression (fold-enrichment = 1.29, *P*_FDR_ = 4.03 × 10^–6^), and cellular macromolecule biosynthetic process (fold-enrichment = 1.29, *P*_FDR_ = 7.86 × 10^–6^). Interestingly, uniquely mapped genes in co-methylation module turquoise were enriched in astrocyte and glial cell functions, which aligned with the enrichment analysis result of co-expression module blue.

### Cross-omics Analysis: OUD-Associated Co-methylated Modules and Co-expression Modules

**Figure 4.**
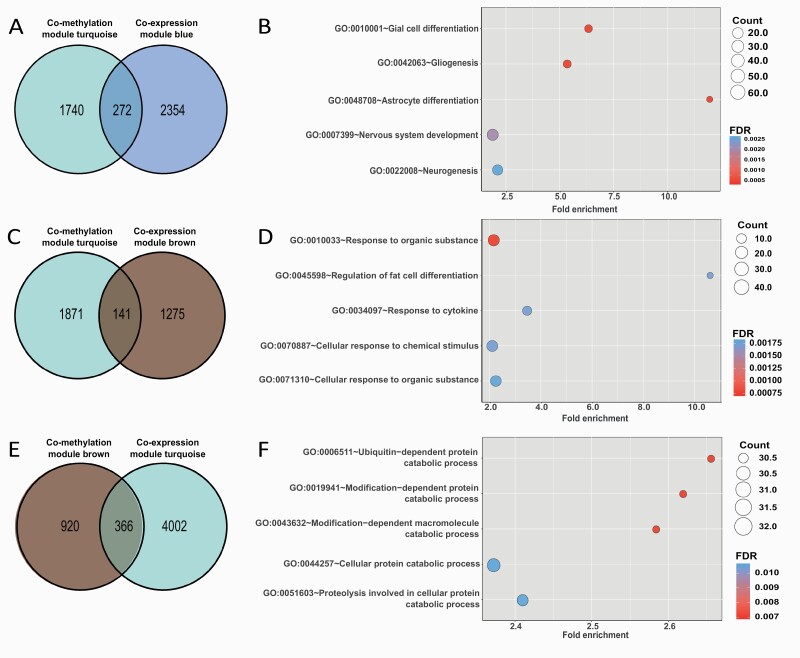
Cross-omics analysis at network module levels highlighted opioid use disorder (OUD)-associated biological processes and gene sets. (A) Venn diagram showing the number of genes that overlap between OUD-associated co-methylation module turquoise and co-expression module blue. (B) Top 5 enriched Gene Ontology (GO) Biological Process (BP) terms for overlapping genes of co-methylation module turquoise and co-expression module blue. (C) Venn diagram showing the number of overlapped genes between OUD-associated co-methylation module turquoise and co-expression module brown. (D) Top 5 enriched GO BP terms for overlapping genes in co-methylation module turquoise and co-expression module brown. (E) Venn diagram showing the number of overlapped genes between OUD-associated co-methylation module brown and co-expression module turquoise. (F) Top 5 enriched GO BP terms for overlapping genes of co-methylation module brown and co-expression module turquoise. FDR, false discovery rate.

Hypergeometric test was performed on the 6 OUD-associated co-methylation modules and 6 OUD-associated co-expression modules to identify modules that overlapped with each other. Mapped genes in the 3 largest co-methylation modules were overrepresented in 6 gene co-expression modules (Figure 4; supplementary Table 7).

**Figure 5.**
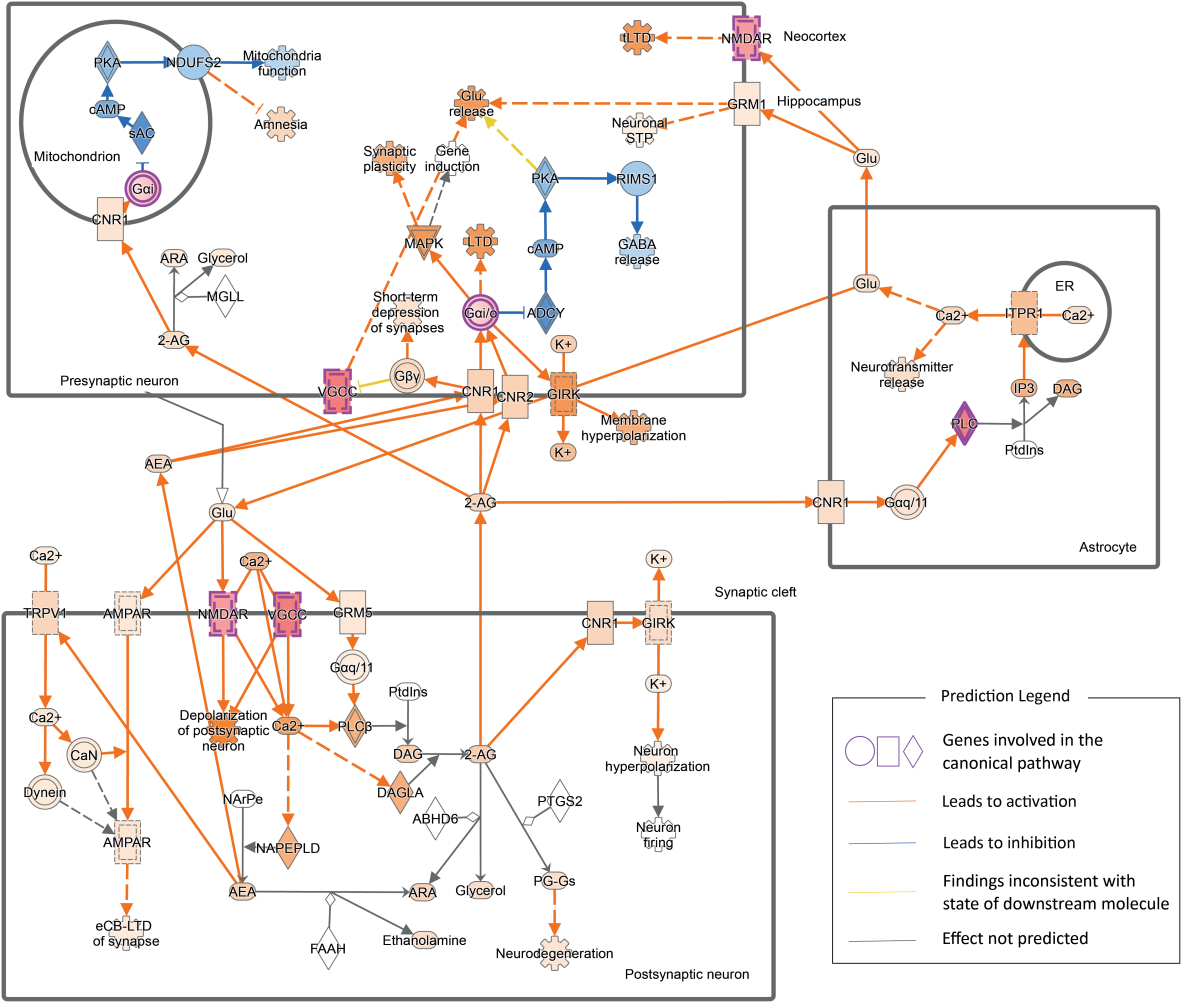
Endocannabinoid neuronal synapse pathway enrichment analysis and predicted regulatory network of overlapping genes between co-methylation module turquoise and co-expression module blue. Highlighted regulatory paths were annotated by ingenuity pathway analysis (IPA).

Of interest, 272 genes overlapped between hypomethylated module turquoise and upregulated co-expression module blue (*P* = 8.42 × 10^–21^) (Figure 4A). The relationship between hypomethylated loci and upregulated genes in these 2 modules aligned with general methylation-expression regulatory patterns. These overlapping genes were enriched in biological processes, including glial cell differentiation, gliogenesis, and astrocyte differentiation (*P*_FDR_ < .001), which aligned with the results of individual enrichment analysis for these 2 modules (Figure 4B; supplementary Tables 2, 4, 8). Then IPA was conducted on the 272 overlapping genes to explore enriched canonical pathways. As shown in Figure 5, the overlapping genes were significantly enriched in endocannabinoid neuronal synapse pathway (*P* = .03, z-score = 2.00). In addition, the 272 overlapping genes were significantly enriched in opioid signaling canonical pathways (supplementary Figure 2). A total 141 overlapping genes were identified between hypomethylated module turquoise and upregulated co-expression module brown (*P* = 1.78 × 10^–12^) (Figure 4C; supplementary Table 9). Enrichment analysis revealed significantly enriched pathways involved in response to organic substance, regulation of fat cell differentiation, and response to cytokine (*P*_FDR _< _ _.005) (Figure 4D). Mapped genes in hypomethylated module brown also significantly overlapped with genes in upregulated co-expression module blue (*P* = 2.06 × 10^–19^) and module brown (*P* = 1.28 × 10^–12^). Moreover, 241 genes in hypermethylated module blue were overrepresented in downregulated co-expression turquoise (*P* = 6.45 × 10^–119^). However, there were no significant functional enrichments in those overlapping genes.

Interestingly, some relationships between co-methylation modules and co-expression modules did not follow canonical methylation-gene expression regulatory patterns. For example, 366 overlapping genes were observed between the hypomethylated module brown and downregulated co-expression module turquoise (*P* = 1.07 × 10^–7^) (Figure 4E). Overall, enriched biological processes for these paradoxical module pairs included catabolic processes and metabolic processes (Figure 4F; supplementary Table 10).

### Cross-omics Analysis: Gene Expression and Methylation Regulation

**Figure 6.**
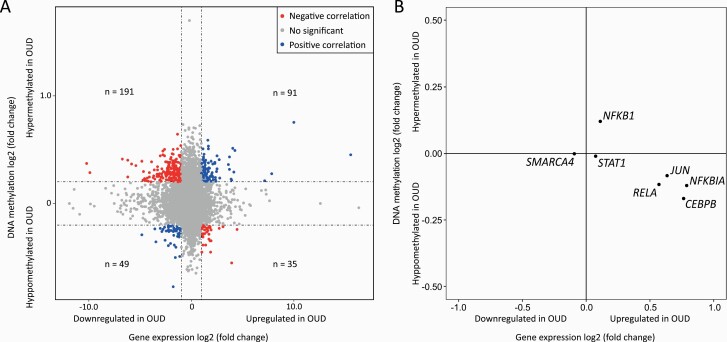
Cross-omics analysis of DNA methylation and gene expression. (A) Correlation analysis between gene expression and DNA methylation changes. The x-axis is the log2 fold change of gene expression between opioid use disorder (OUD) subjects and non-psychiatric controls. The y-axis is the log2 fold change of DNA methylation change of mapped genes. Subgroup 1 contains 91 genes; subgroup 2 contains 191 genes; subgroup 3 contains 49 genes; and subgroup 4 contains 35 genes. (B) Gene expression and DNA methylation changes of potential transcription regulators of 91 genes in subgroup 1. The x-axis is the log2 fold change of gene expression between OUD subjects and non-psychiatric controls. The y-axis is the log2 fold change of DNA methylation change of the mapped genes.

To further explore the correlation between OUD-associated gene expression and methylation changes, we conducted an integrative analysis based on the DEG and DMP analyses (supplementary Figure 3). In total, 787 949 methylation probes were mapped to 16 986 genes. Genes with |expression log2 fold change| < 1 or |median methylation log2 fold change| < 0.2 were removed. After filtering, 1 outlier gene was also removed. As shown in Figure 6A, 366 genes were identified with high DNA methylation and gene expression variations. These genes could be divided into 4 subgroups based on DNA methylation and expression levels: (1) 91 genes hypermethylated and upregulated; (2) 191 genes hypermethylated and downregulated; (3) 49 genes hypomethylated and downregulated; and (4) 35 genes hypomethylated and upregulated (Figure 6A). In general, our results revealed that more genes followed the trend that increased DNA methylation correlate downregulates gene expression (1.61-fold), aligning with the general methylation-gene expression regulatory patterns (Suzuki and Bird, 2008).

Pathway enrichment analysis was next performed using IPA to explore potential transcription regulators for each subgroup of genes. Of interest, IPA of 91 genes in the first subgroup (Figure 6A) showed that 7 immune-related transcription regulators (CEBPB, JUN, NFKB1, NFKBIA, RELA, SMARCA4, and STAT1) contribute to the positive association between hypermethylation and upregulated gene expression (Figure 6B; supplementary Figure 4). Interestingly, 5 of these 7 transcription regulators were hypomethylated and upregulated. Among the 191 genes in the second subgroup (Figure 6A), 3 upstream transcription regulators, HNF1A, IRF7, and SOX11, were revealed. No significant transcription regulators were discovered for the genes of the third or the fourth subgroup.

## Discussion

In the present study, we explored gene expression and DNA methylation alterations in postmortem brain samples between 22 OUD subjects and 19 non-psychiatric controls. We identified 6 gene co-expression modules and 6 co-methylation modules that were significantly associated with OUD trait. Functional enrichment analysis of both co-expression and co-methylation modules revealed gene pathways enriched in signaling, nervous system development, cellular response to stimulus, and astrocyte-related metabolic process, transport, and differentiation. In particular, this analysis revealed that astrocyte processes might be involved in OUD pathogenesis. Subsequently, cross-omics analyses revealed immune-related transcription regulators aligned with methylation-gene expression regulatory patterns. These findings suggested that transcription regulator-related networks may play some critical roles in OUD pathogenesis.

Functional enrichment analyses identified astrocyte and glial cell-related genes that are regulated by methylation-gene expression relationships. It is noteworthy that overlapping genes between the co-methylation module turquoise and co-expression module blue highlighted astrocyte and glial cell differentiation, gliogenesis, and nervous system development, which were also supported by individual-level module enrichment analysis (supplementary Tables 2, 4, 8). Among the brain cell functional analysis (Goudriaan et al., 2014), hub genes of co-expression module blue showed cell-type enrichment in astrocyte transport, trafficking, and metabolism functions (supplementary Figure 1). Moreover, IPA results revealed astrocytic function and the endocannabinoid neuronal synapse pathway that might play a role in OUD pathogenesis (Figure 5). These results align with previous studies showing morphine induces astrocyte differentiation in mice (Xu et al., 2016).

Moreover, functional enrichment analysis on overlapping genes of hypomethylated module turquoise and upregulated co-expression module brown revealed processes involved in response to organic substances and cytokines (Figure 4). Cytokine activation is known to modulate opioid receptor signaling and is likely involved in the development of addictive disorders like OUD (Law et al., 2000; Eidson and Murphy, 2019).

Regulatory networks that involve transcription regulators were identified in our cross-omics analysis. As shown in Figure 6, we identified 91 hypermethylated and upregulated genes in the first subgroup, a finding that may seem inconsistent with the paradigm of methylation-expression regulation. IPA suggested that several immune-related translational regulators (CEBPB, JUN, NFKB1, NFKBIA, RELA, SMARCA4, and STAT1) were involved, all of which were hypomethylated and upregulated (Figure 6B). The regulatory relationships of these transcription regulators aligned with the general methylation-expression regulatory patterns. This finding suggests upstream transcription regulators may override the suppressive effects of hypermethylation on the target genes, although individual-level and single-cell level conclusions remain investigated (supplementary Figure 4). In the second subgroup, their potential upstream regulators (HNF1A, IRF7, and SOX11) could downregulate target genes. Thus, the effects of transcription regulators are in line with the hypermethylation effects on their targets. Alternatively, the MethylationEPIC kit measures both 5-hydroxymethylcytosine and 5-methylcytosine, but it does not distinguish between them. 5-Hydroxymethylcytosine promotes gene transcription, the opposite effect of 5-methylcytosine, thereby having a functional demethylation effect (Ponnaluri et al., 2017).

The results of the current study should be interpreted carefully due to several limitations. First, although the present work had one of the largest sample sizes compared with the previously published OUD studies, effect size of substance use disorders in genetic studies is often small, which hinders statistical power (Cecil et al., 2016; Dai et al., 2020; Stamatovich et al., 2020). Second, the methylation and RNA-seq data were not perfectly matched. Complete gene expression and DNA methylation data were available for 29 out of 41 samples, while 12 samples only have gene expression data or DNA methylation data. Considering the limited statistical power, we did not exclude samples with only 1 level of data. Thus, current findings only reflected the shared signals among samples, not matched individual omics comparisons. Third, we only considered methylation probes in promoter and TSSs of genes in the current study. Only 11 917 significant DMPs located in TSSs were included in network analysis, which might not fully represent each gene’s methylation level. Methylation probes located in non-TSS regions were not studied in the current project, but accumulating evidence suggests diverse methylation functions in the gene body (Lister et al., 2009; Jones, 2012; Mallik and Zhao, 2017). Further studies are needed to examine the role of methylation in non-TSS regions and better understand the effects of methylation on OUD pathology. Lastly, as the significance threshold was loosened in some analyses due to relatively small sample size, we applied several different multiple testing correction criteria. In WGCNA, we used *P*_FDR_ < .1 as the threshold for OUD-associated co-expression and co-methylation cluster selection. We will continue collecting more samples and integrating other omics data (e.g., genomic, proteomic, and single-cell level data). Further validation on protein expression or clinical features will support our findings. Future studies will also investigate the relationship between DNA methylation and gene expression alterations in the brain and blood, which could lead to the identification of candidate biomarkers.

## Conclusions

In conclusion, we developed a framework to conduct an integrative analysis of 41 OUD postmortem brain samples at DNA methylation and gene expression levels. We identified sets of genes, transcription regulators, co-expression and co-methylation modules, and biological pathways associated with OUD, highlighting the important roles of astrocytes and glial cells. Through cross-omics analysis, we observed both standard and paradoxical DNA methylation and gene expression relationships, suggesting there may be other complex gene regulatory mechanisms involved in these expression patterns, such as regulation of upstream transcription regulators. Taken together, the current findings suggested that regulation of astrocyte and glial cells involved molecular mechanisms might constitute promising targets for OUD treatment.

## Supplementary Material

pyab043_suppl_Supplementary_Figure_1

pyab043_suppl_Supplementary_Figure_2

pyab043_suppl_Supplementary_Figure_3

pyab043_suppl_Supplementary_Figure_4

pyab043_suppl_Supplementary_Tables

## Statement of Interest

The funders had no role in the study design, data collection and analysis, decision to publish, or preparation of the manuscript.
