# Supplementary figures and images for: Genome-Wide Correlation of DNA Methylation and Gene Expression in Postmortem Brain Tissues of Opioid Use Disorder Patients
Source: Int J Neuropsychopharmacol. 2021 Jul 2;24(11):879–91. doi: 10.1093/ijnp/pyab043 (PMC8598308; doi:10.1093/ijnp/pyab043)

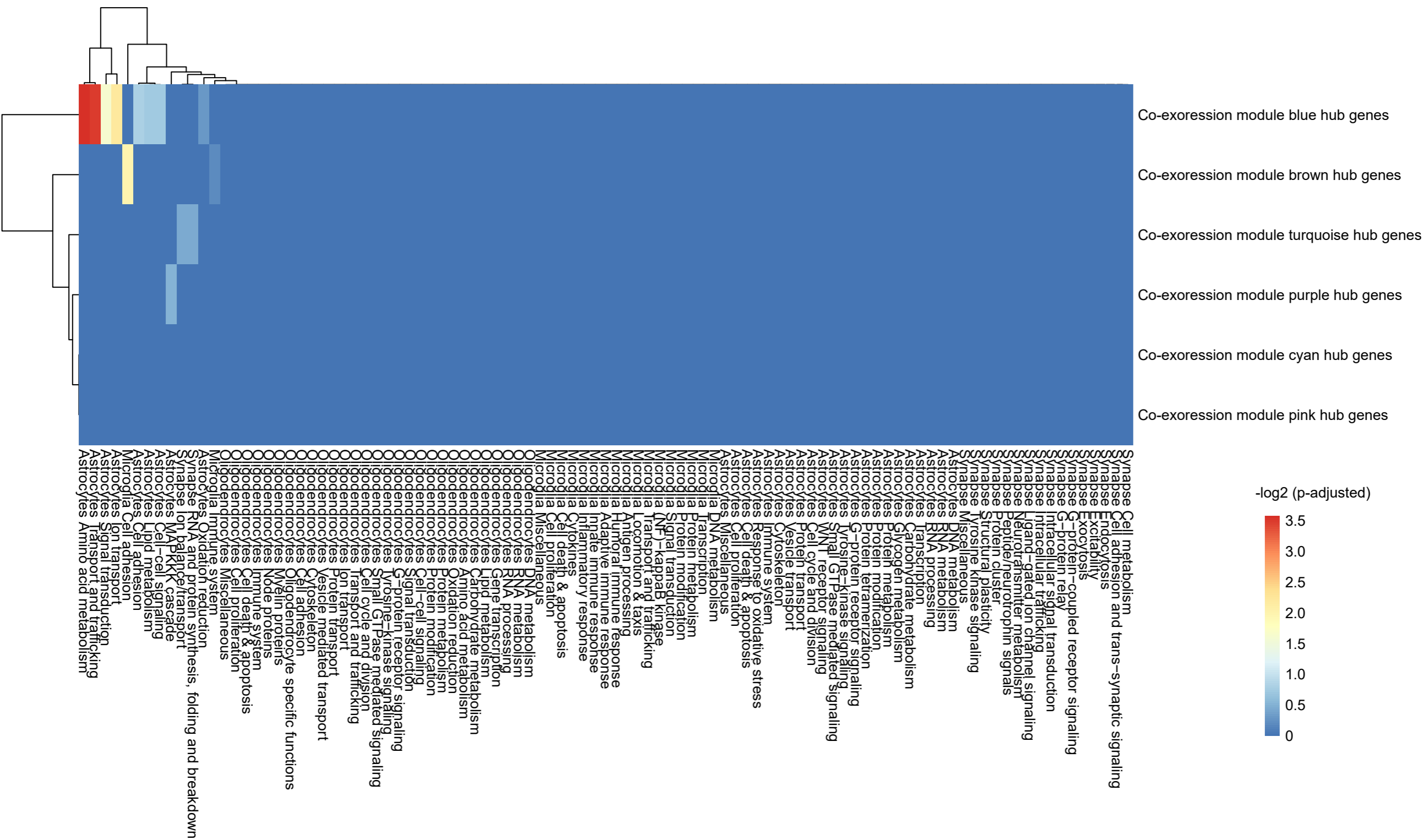

Supplement: pyab043_suppl_Supplementary_Figure_1 [file pyab043_suppl_supplementary_figure_1.pdf]

**A** Differentially expressed genes analysis

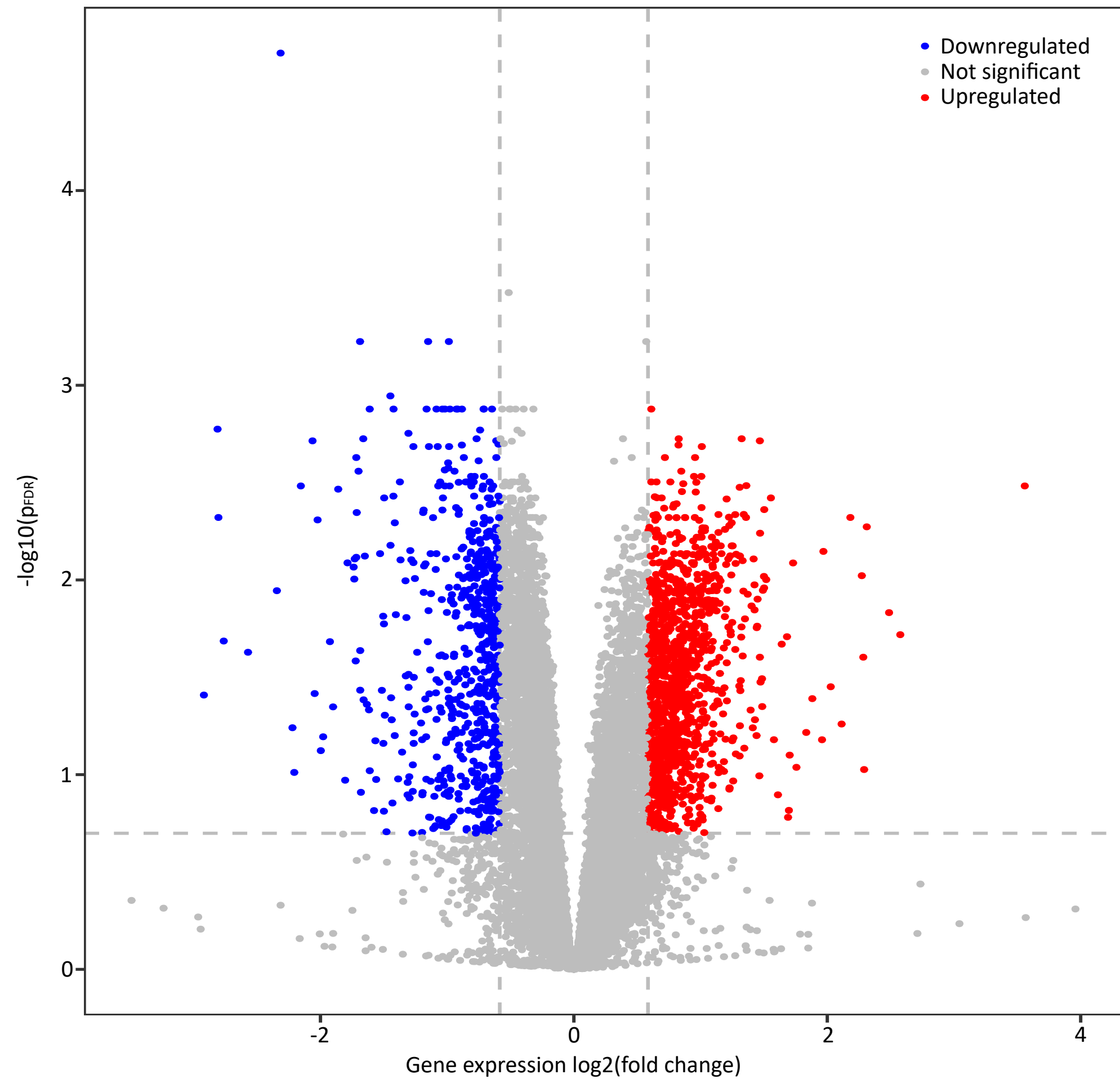

**B** Differentially methylated probes analysis

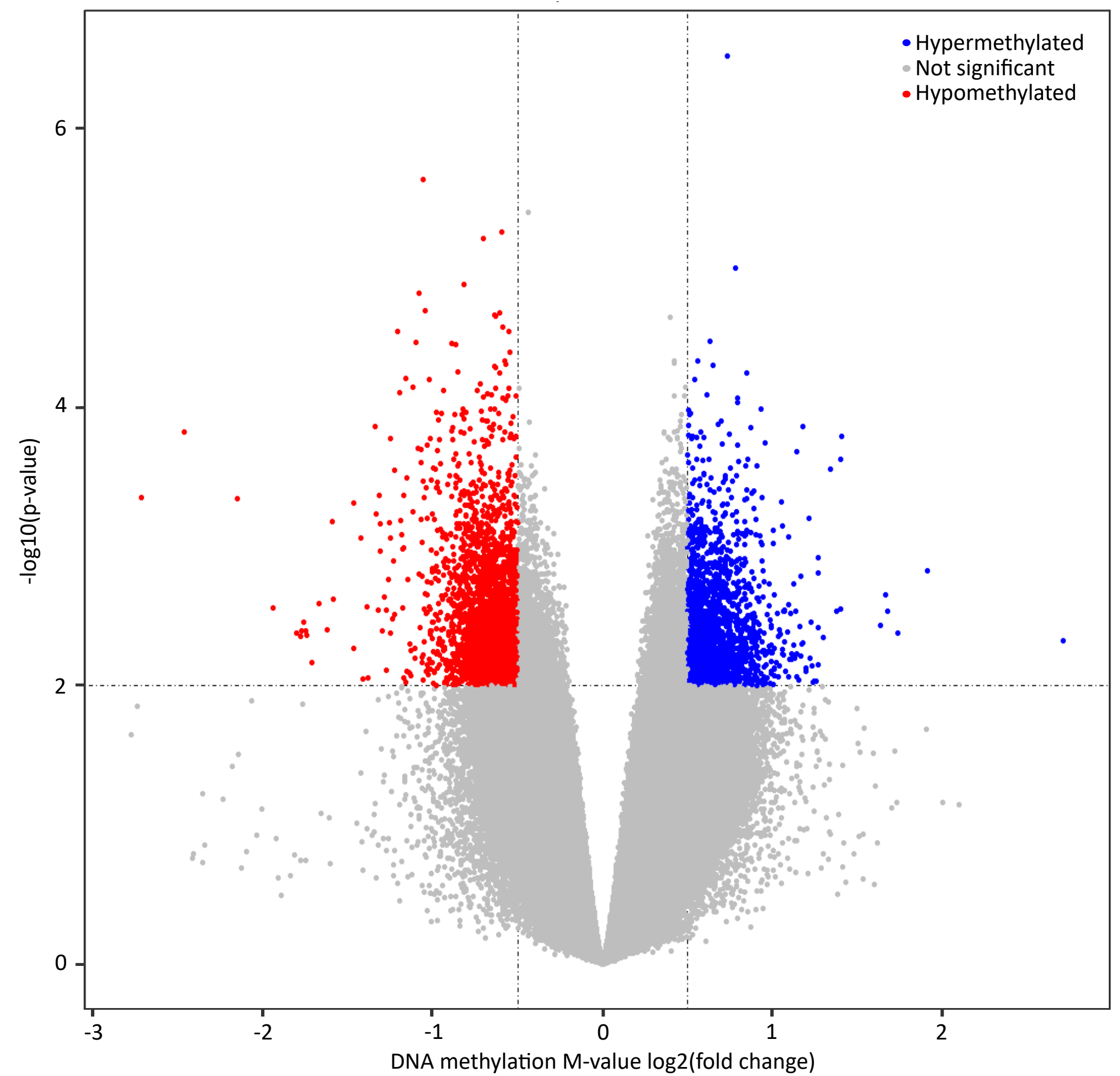

Supplement: pyab043_suppl_Supplementary_Figure_3 [file pyab043_suppl_supplementary_figure_3.pdf]

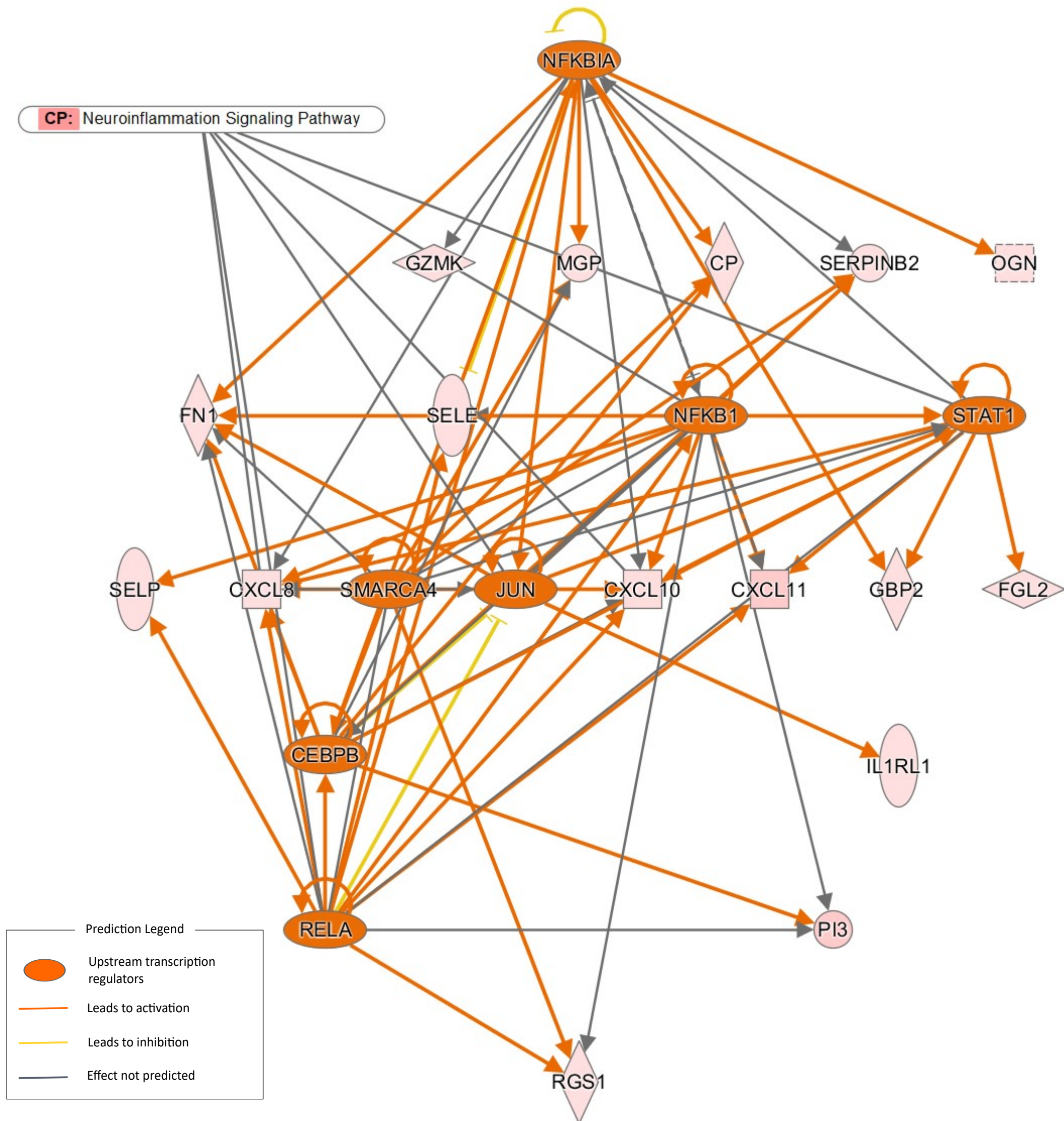

Supplement: pyab043_suppl_Supplementary_Figure_4 [file pyab043_suppl_supplementary_figure_4.pdf]
