# Supplementary material for: Genome-Wide Correlation of DNA Methylation and Gene Expression in Postmortem Brain Tissues of Opioid Use Disorder Patients
Source: Int J Neuropsychopharmacol. 2021 Jul 2;24(11):879–91. doi: 10.1093/ijnp/pyab043 (PMC8598308; doi:10.1093/ijnp/pyab043)
Supplement: pyab043_suppl_Supplementary_Figure_2 [file pyab043_suppl_supplementary_figure_2.pdf]

Opioid Signaling Pathway

Opioid receptors belong to the G protein-coupled receptor family, characterized by a seven-transmembrane span structure.
Opioid receptors are expressed on primary sensory neurons in pain-modulating descending pathways,

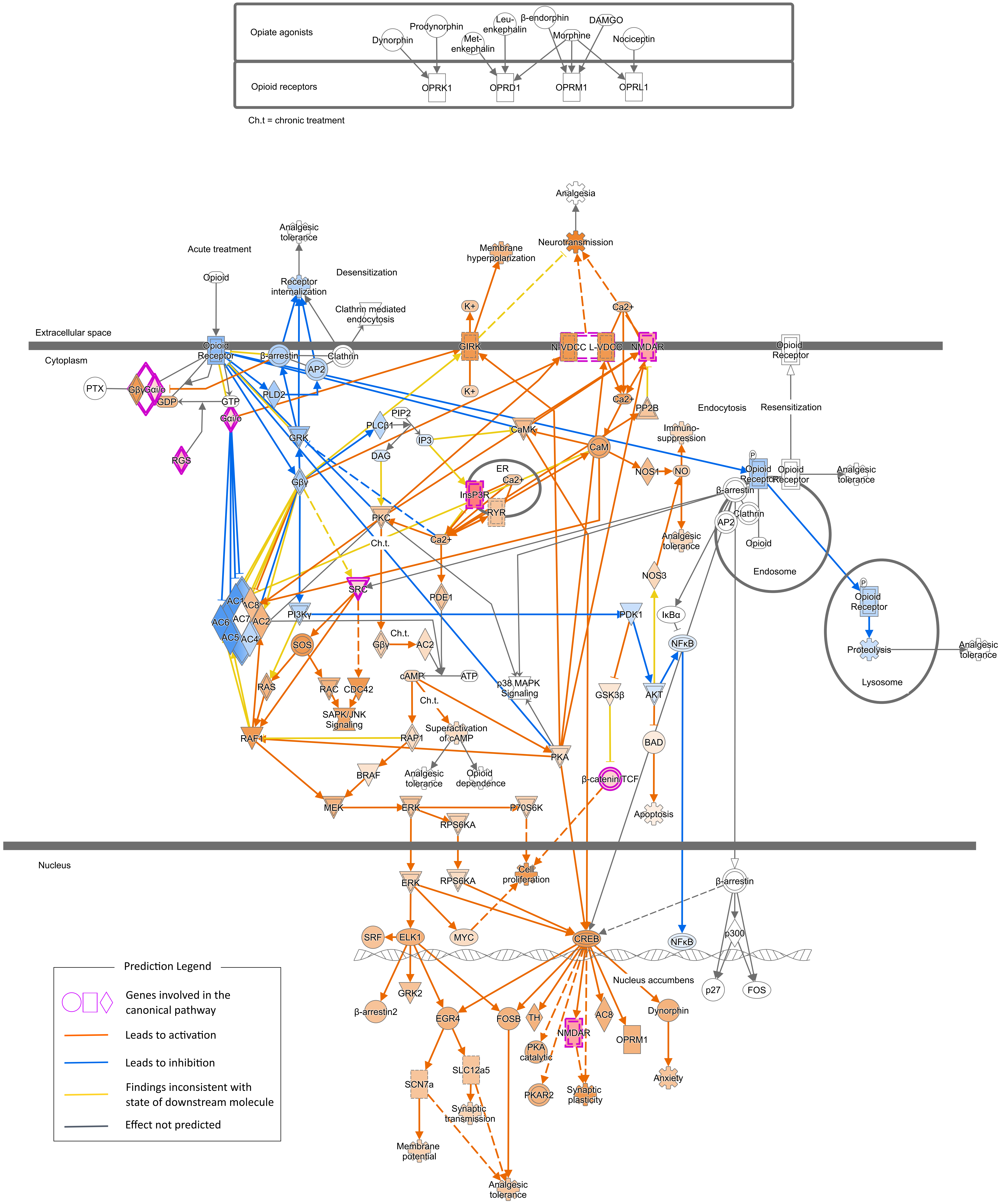

© 2000-2021 QIAGEN. All rights reserved.
